# Right Drug in the Wrong Place: The Effects of Inadvertent Intrathecal Tranexamic Acid

**DOI:** 10.7759/cureus.72661

**Published:** 2024-10-29

**Authors:** Daniela Simões Ferreira, Catarina Dias, Liliana Costa, Margarida Bettencourt

**Affiliations:** 1 Anesthesiology, Unidade Local de Saúde da Região de Aveiro, Aveiro, PRT

**Keywords:** administration errors, anesthesia for spine surgery, intrathecal complication, intravenous tranexamic acid, perioperative blood loss, postoperative seizures, topical tranexamic acid

## Abstract

Tranexamic acid (TXA) is an antifibrinolytic drug widely used to reduce blood loss in major surgeries and trauma patients, thus reducing morbimortality. In recent years, clinical indications for TXA have expanded, including many off-label uses. This broad use has led to an increased incidence of reported side effects and administration errors with serious neurological and cardiovascular outcomes, such as seizures, myoclonus, and arrhythmias. This case report describes a 75-year-old female, American Society of Anesthesiologists (ASA) physical status III, who underwent lumbar spine surgery and received topical TXA for hemostatic control despite intraoperative incidental dural tears. Postoperatively, the patient experienced severe back pain, perianal burning, and painful lower limb myoclonus, prompting emergency surgery to rule out surgical complications. Despite no structural issues being identified, her symptoms persisted, requiring reintubation, sedation, and ICU admission. On the first postoperative day, no further pain or myoclonus was present with analgosedation and mechanical ventilation weaning. The patient's clinical condition improved gradually, being discharged one week later with no further complaints or neurological sequelae. TXA reduces inhibitory neurotransmission of γ-Aminobutyric acid type A (GABA-A) and glycine receptors with increased neuronal excitability and potential proconvulsant properties. This mechanism of action is responsible for the clinical features of acute neurotoxicity with imbalanced inhibitory-excitatory motor, sensory, and autonomic neurotransmission following intrathecal TXA. This case report intends to increase awareness for early recognition of the neurotoxic effects following inadvertent intrathecal TXA. The authors are unaware of other reports regarding this clinical feature as a complication from spinal topical use, enhancing the importance of dural integrity before considering topical TXA administration. Any dural tear could allow TXA to enter the intrathecal space, potentially leading to severe neurotoxic effects. In conclusion, further studies are required to evaluate the safety, efficacy, and clinical indications for topical TXA, particularly in spine surgery, to provide a safer use of this promising drug.

## Introduction

Tranexamic acid (TXA) is a synthetic lysine analog that acts as an antifibrinolytic drug by competitively binding to plasminogen and plasmin, thus reducing the degradation of fibrin-containing blood clots [1]. Once activated, plasmin can have both stimulatory and inhibitory effects on platelets, playing a crucial role in hemostasis and thrombosis. Additionally, plasmin is known to have pro-inflammatory and immunosuppressive effects through monocyte activation and cytokine production. TXA blocks plasmin formation, thus exhibiting an immune system modulatory effect on inflammatory cytokine levels and innate immune cell activation, with subsequent reduced systemic inflammatory response, in addition to the effect on reducing blood loss [2].

Antifibrinolytic drugs, including TXA and ɛ-aminocaproic acid, are considered safe and affordable drugs with few adverse effects. Their clinical indications have rapidly expanded in the last decade, particularly after the 2010 Clinical Randomization and Antifibrinolytic in Significant Hemorrhage (CRASH-2) trial of TXA in adult trauma patients with significant bleeding [3, 4]. Further evidence from various prospective randomized studies and meta-analyses recommends TXA use as prophylaxis or therapeutical treatment to reduce perioperative and traumatic blood loss despite the increased incidence of seizures reported in the early postoperative period [1, 5]. These studies demonstrated a mean reduction of 34% in blood loss concerning major surgeries and 39% in the requirement for blood transfusions in the postoperative period in cardiac, obstetric, and urologic surgery [4]. Its efficacy has also been assessed in knee and hip arthroplasty in placebo-controlled trials, with recommended pre-incision intravenous administration [2]. Regarding spine surgery, its use remains mainly in off-label conditions due to the poor methodological quality of the clinical trials and heterogeneity regarding the type of surgery, drug dose, and administration regimen [5]. However, as evidence suggests a decrease in blood loss and transfusion rates with minimal side effects, it continues to be used in major spine surgery with high bleeding risk (>30% total estimated blood volume) or multilevel spinal fusion [5]. Concerning administration routes, topical TXA is a promising strategy that favorably reduces blood loss, with fewer systemic side effects than intravenous TXA, such as seizures and thromboembolism. However, evidence on safety, efficacy, and dose of topical administration is still not robust [5].

The broad use of TXA led to increased reports of administration errors associated with serious neurological and cardiovascular outcomes. Several cases of inadvertent spinal administration of TXA instead of commonly used local anesthetics due to ampule similarity have been reported in recent years and have offered additional insights into the clinical features of TXA-associated seizures [1, 6]. Clinical presentation included perianal burning, severe lower back pain, and lower limb myoclonus, potentially progressing to generalized seizures and refractory ventricular arrhythmias [7-9]. Intrathecal TXA is associated with death or permanent harm in more than 50% of patients. On the other hand, in the cases of patients who survived, there were no reported neurological or cardiovascular sequelae [7].

## Case presentation

In this case report, a 75-year-old female, American Society of Anesthesiologists (ASA) physical status III, diagnosed with lumbar spinal stenosis, underwent spinal surgery with laminectomy and vertebral fixation at L4-L5 level. The patient had a medical history of hypertension, dyslipidemia, obesity grade I (BMI of 32 kg/m2), peripheral arterial disease, and venous insufficiency. Preoperative evaluation excluded any known allergies, and instructions for medication management were provided. Assessment of the patient's airway revealed no expected difficulties in airway management. Preoperative laboratory tests were within normal range values and EKG in sinus rhythm. Cardiac auscultation revealed a holosystolic murmur grade 4/6, and the patient reported complaints of dyspnea with mild efforts, so a preoperative cardiology consultation was solicited to determine the need for additional testing preoperatively. A transthoracic echocardiogram was performed, revealing moderate to severe mitral regurgitation due to posterior mitral leaflet prolapse, with no signs of pulmonary hypertension. There was no contraindication from the cardiology assistant for the surgery proposed.

**Figure 1:**
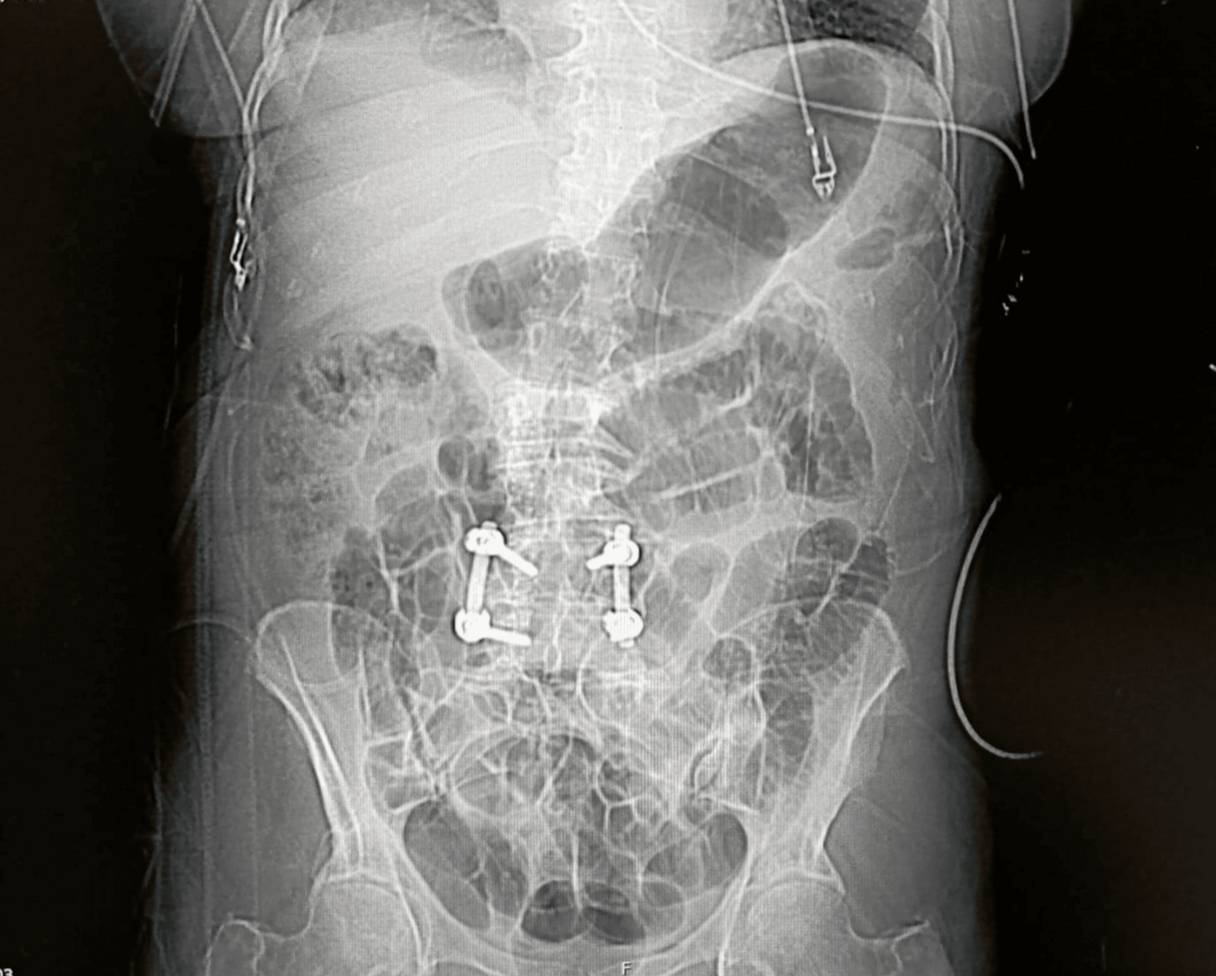
Postoperative spinal CT scan Thoracolumbar coronal CT image showing spinal fusion and instrumentation at L4-L5.

**Figure 2:**
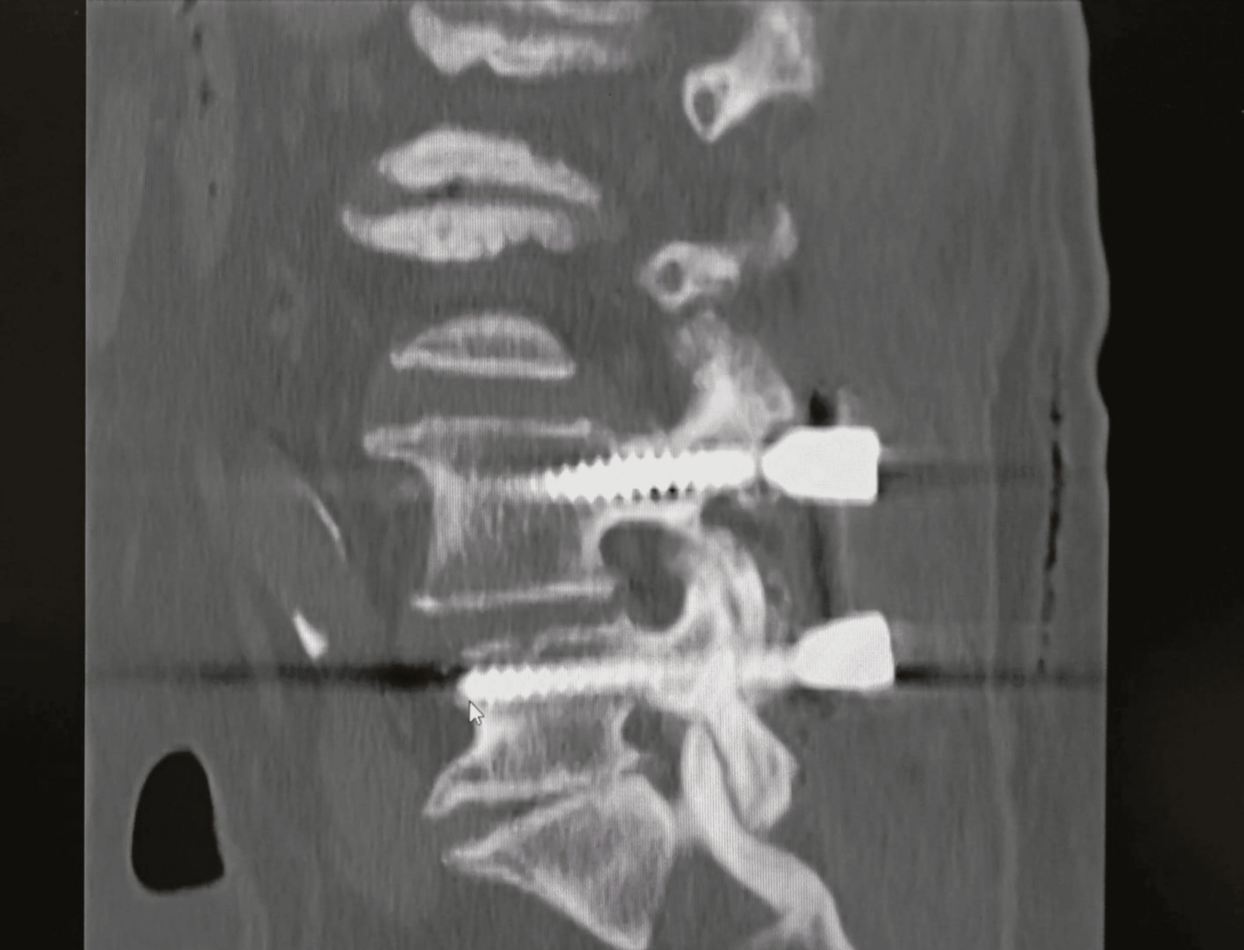
Postoperative spinal CT scan Lumbar sagital CT image showing spinal fusion and instrumentation. Alignment of the spine and placement of the screws seem to be central and symmetrical, which suggests proper positioning.

**Figure 3:**
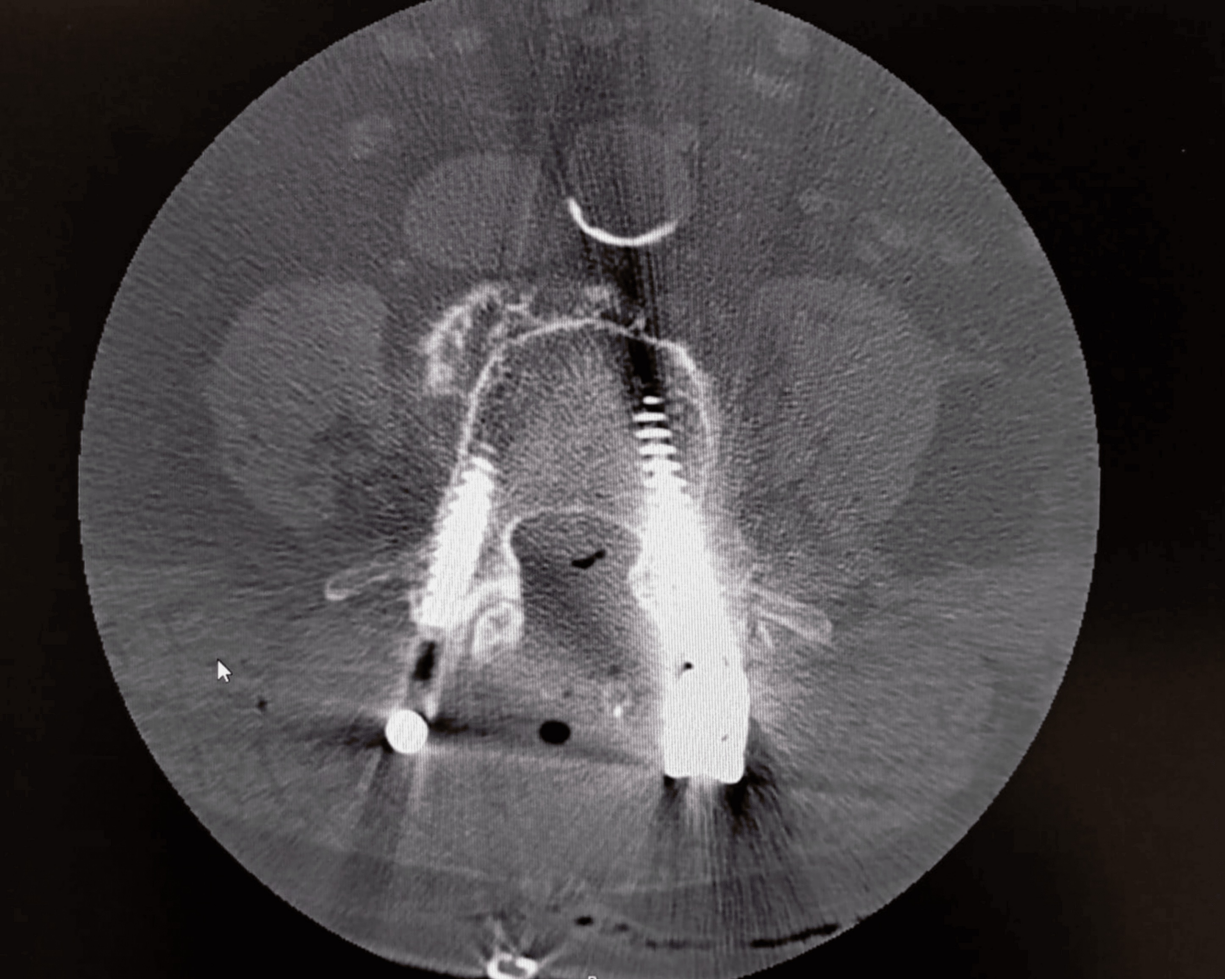
Postoperative spinal CT scan Cross-sectional (axial) CT scan of the lumbar spine with the two screws visible on either side of the vertebra, inserted bilaterally into the pedicles, suggesting proper positioning.

A balanced general anesthesia was induced, and the patient was placed in a prone position for a posterior approach, maintaining respiratory and hemodynamic stability throughout. In the intraoperative course, there were two incidental dural tears during laminectomy maneuvers, which were sutured, and before wound closure, 1g of topical TXA was administered for hemostatic control. Emergence from anesthesia was uneventful, and the patient was transferred to the post-anesthesia care unit. A few minutes later, the patient reported perianal burning, severe lower back pain, and subsequent painful lower limbs myoclonus were observed. Due to suspicion of an immediate postoperative complication, namely a spinal hematoma, the patient was immediately transferred to the operating room and submitted to emergency surgery, with no significant findings for the previous symptoms. During emergence from anesthesia, the patient's blood pressure and heart rate increased to 197/98 mmHg and 129 beats per minute. Painful myoclonic movements persisted despite intravenous midazolam 4mg administration, requiring reintubation with sedation and neuromuscular blockade with admission to the intensive care unit (ICU) for further care and investigation. A lumbar CT scan ruled out structural underlying causes (Figures 1-3).

Postoperative day arterial blood gas analysis revealed metabolic acidosis (pH 7,28, PaO2 142, PaCO2 38, HCO3- 18, SpO2 97%) and mild hypokalemia (K+ 3,1), which was corrected with 40mEq of intravenous potassium chloride. Hematological, renal, and hepatic evaluation revealed no major abnormalities (Tables 1, 2).

**Table 1:** Postoperative arterial blood gas analysis. Postoperative arterial blood gas analysis with metabolic acidosis, mild hypokalemia, hypocalcemia, hyperlactacidemia, and anemia. Ca2+ - calcium; Cl- - chloride; Hb - hemoglobin; HCO3- - bicarbonate; Htc - hematocrit; K+ - potassium; Na+ - sodium; PaCO2 - partial pressure of carbon dioxide; PaO2 - partial pressure of oxygen; SpO2 - oxygen saturation

Parameters	Patient values	Reference range
pH	7.28	7.35 – 7.45
PaO2 (mmHg)	142	75.0 – 105
PaCO2 (mmHg)	38	35.0 – 45.0
HCO3- (mmol/L)	18	22.0 – 28.0
SpO2 (%)	95	95.0 – 99.0
K^+^ (mmol/L)	3.1	3.7 – 4.7
Na^+^ (mmol/L)	140	136 – 146
Cl^-^ (mmol/L)	102	98 - 110
Ca^2+^ (mmol/L)	1.12	1.15 – 1.30
Glucose (mg/dL)	126	70-100
Lactate (mmol/L)	2.1	0.5 – 2.0
Hb (g/dL)	9.0	12-15
Htc (%)	29.6	36-44%

**Table 2:** Postoperative laboratory investigations Postoperative blood tests were performed in the intensive care init (ICU) revealed anemia and thrombocytopenia. Coagulation, renal, and hepatic evaluation revealed no major abnormalities. ALP - alkaline phosphatase; ALT - alanine transaminase; APTT - activated partial thromboplastin time; AST - aspartate transaminase; BUN - blood urea nitrogen; Cl- - chloride; Cr - creatinine; GGT - gamma-glutamyl transferase; Hb - hemoglobin; Htc - hematocrit; INR - international normalized ratio; K+ - potassium; LDH - lactate dehydrogenase; Na+ - sodium; PCR - polymerase chain reaction; PT - prothrombin time; RBC - red blood cells; WBC - white blood cells.

Parameters	Patient values	Reference range
Hb (g/dL)	9.4	12.0 - 16.0
Htc (%)	27.8	40 - 54%
RBC (x10^12^/L)	2.91	4.50 – 6.00
WBC (x10^9^/L)	9.6	4.50 – 11.50
Platelets (x10^9^/L)	182	150 - 350
Glucose /mg/dL)	86	74 - 106
BUN (mg/dL)	42	19 - 49
Cr (mg/dL)	0.55	0.8 – 1.3
K^+^ (mEq/L)	3.45	3.5 – 5.0
Na^+^ (mEq/L)	142.95	135 – 145
Cl^-^ (mEq/L)	107.54	95.0 - 110.0
AST (U/L)	87	4 – 43
ALT (U/L)	38	4 - 43
LDH (U/L)	247	120 - 246
ALP (U/L)	79	25 - 100
GGT (U/L)	33	5 - 73.0
Total Bilirubin (mg/dL)	0.57	0.2 – 1.1
PCR (mg/dL)	7.01	< 0.50
INR	1.0	0.9 - 1.2
PT (s)	11.2	11.7 - 15.3
PTT (s)	22.5	25.0-35.0

Mechanical ventilation and sedoanalgesia weaning were initiated throughout the second postoperative day after analytical improvement, with no myoclonus detected. The patient was extubated and had no further complaints of perianal burning or lower limb pain and no memory of the postoperative events. The patient's clinical condition improved gradually and she was discharged seven days later from the hospital with no further complaints or neurological sequelae during the first year of follow-up. 

## Discussion

Tranexamic acid has been widely used in recent years as a prophylactic treatment to reduce perioperative blood loss in major surgery and trauma patients, thus reducing the requirement for blood transfusions and lowering morbidity and mortality associated with hemorrhage [3]. This broad introduction has also led to an increased reported incidence of seizures. Retrospective studies have identified several risk factors for TXA-associated seizures, such as higher doses, female gender, increased age, and poor overall health with renal dysfunction or prior neurological and cardiovascular disorders. Other important risk factors include the type and duration of surgery [1].

The potential proconvulsant properties of TXA are concerning, and increasing evidence suggests that TXA increases the excitability of neuronal networks from reduced inhibitory neurotransmission of γ-Aminobutyric acid type A (GABA-A) and glycine receptors by acting as a competitive antagonist [1, 3]. This imbalanced inhibitory-excitatory motor, sensory, and autonomic neurotransmission is responsible for the clinical features previously mentioned. Therefore, myoclonic movements may serve as a warning sign of impeding seizures, and uncontrolled sympathetic output can lead to tachycardia, hypertension, and tachyarrhythmias with a risk of refractory ventricular fibrillation. Some studies suggest that general anesthetics, including propofol and halogenated inhalational anesthetics, should be considered as first-line treatment by acting as positive allosteric modulators of glycine receptors, rapidly reversing the TXA inhibition of tonic glycine current [1]. However, many questions and mechanisms remain unanswered.

A recent narrative reviewed 22 case reports of administration errors during spinal anesthesia with accidental intrathecal TXA, exploring its clinical features and proposing strategies to manage subsequent neurological and cardiovascular toxicity. This research showed that permanent harm or mortality was reported in more than 50% of cases [6]. Acute lower limbs or back pain, seizures, tachycardia, hypertension, and arrhythmias were the most common clinical features of intrathecal TXA, as observed in this patient following topical spinal use, leading to suspicion of TXA neurotoxicity due to intrathecal penetration through the incidental dural tears. The management of these features is mainly supportive and symptomatic but requires high suspicion and prompt clinical diagnosis since it's a life-threatening emergency. 

Topical TXA administration in patients undergoing spine surgery favorably reduces perioperative blood loss, hospitalization length, and systemic side effects associated with intravenous TXA, such as seizures and thromboembolism [10]. Recent meta-analyses have also emphasized the advantages of topical TXA compared with intravenous; however, they focused on articles published before 2019, and 70% of the literature on topical administration was published afterward [10-12]. Furthermore, prior meta-analyses included high heterogeneity randomized controlled trials (RCT) with limited results.

## Conclusions

This case report intends to increase awareness of safety in regard to TXA use and the importance of high suspicion and early recognition of the warning signs of potentially fatal outcomes due to inadvertent intrathecal TXA. In recent years, there has been an increase in reported accidental spinal administration of TXA, but the authors are unaware of other reports regarding this complication with topical use in spine surgery. This case suggests that the use of topical TXA in spine surgery should be reserved for surgeries where dural integrity is assured in order to prevent intrathecal penetration of TXA and the associated risk of neurotoxicity such as seizures, myoclonus, or permanent neurological damage. 

In conclusion, evidence suggests that the safety and efficacy of topical TXA in spine surgery is still not robust, and further RCTs are required to establish the clinical indications and contraindications for topical administration, the optimal therapeutic dose, and the pharmacodynamics of topical TXA, thus providing a safer use of this promising strategy.
